# Braking Bad: Stopping Translation in Hard Times

**DOI:** 10.1371/journal.pbio.1001867

**Published:** 2014-05-20

**Authors:** Roland G. Roberts

**Affiliations:** Public Library of Science, Cambridge, United Kingdom

A typical bacterial cell may contain tens of thousands of ribosomes, each one a tiny but intricate clamshell structure of two subunits, one large and one small. When times are good, they spool along mRNAs, translating each codon into an amino acid that's incorporated into a growing polypeptide to make the thousands of different proteins needed for cellular life. Between the two halves of the clamshell is the heart of the machine, where the tRNA adaptors—anticodon at one end and amino acid tagged onto the other—dock into the ribosome, find their match on the mRNA, and impart their amino acid cargo to the growing chain.

That's when all is going well, but what happens when times get tough and that crucial supply of amino acids dries up? Making proteins is an expensive business, and if nutrients are running low, the cell needs to slam on the brakes and concentrate on the basic task of staying alive. This belt-tightening decision is known as the “stringent response”, and is signalled by the so-called “alarmones”, guanosine tetraphosphate and pentaphosphate, collectively named as (p)ppGpp, chemicals made by bacteria in response to low cellular levels of amino acids.

(p)ppGpp is known to inhibit several basic aspects of bacterial physiology, including replication of the genome and transcription of a wide range of genes, mostly related in turn to translation. It also appears directly to inhibit translation itself, but the mechanism by which it does this has been unclear. A paper just published by Boya Feng, Ning Gao, and colleagues in *PLOS Biology* sheds some light on this.

The focus of the study is ObgE, a protein from an atypical branch of the small GTPase family of enzymes. ObgE was known to have a role in the assembly of the large ribosomal subunit, and there was already some evidence that it could bind (p)ppGpp. Small GTPases usually act as cellular switches, changing state according to whether their active site is occupied by GTP or GDP. However, (p)ppGpp is closely related to GTP and GDP, differing only in the addition of a pyrophosphate group, so a GTPase like ObgE makes an intriguing candidate for a (p)ppGpp receptor.

The authors first show that ObgE can bind to the larger of the two ribosomal subunits, and that adding ObgE to intact ribosomes disrupts the clamshell structure into its component halves. This activity depends on the presence of guanosine nucleotides. The authors then dissect ObgE, finding that the rather variable and flexible C-terminus isn't needed for ribosome disruption and that this property resides instead in the highly conserved N-terminal and central GTPase domains.

Following the kinetics of ribosome formation from separate subunits, the authors show that ObgE binds to the large ribosomal subunit, competing with the small subunit and preventing its association with its partner. This function is that of an “anti-association factor”—another protein, IF3, is known to perform a reciprocal anti-association role by binding to the small subunit and competing with the large one.

**Figure 1:**
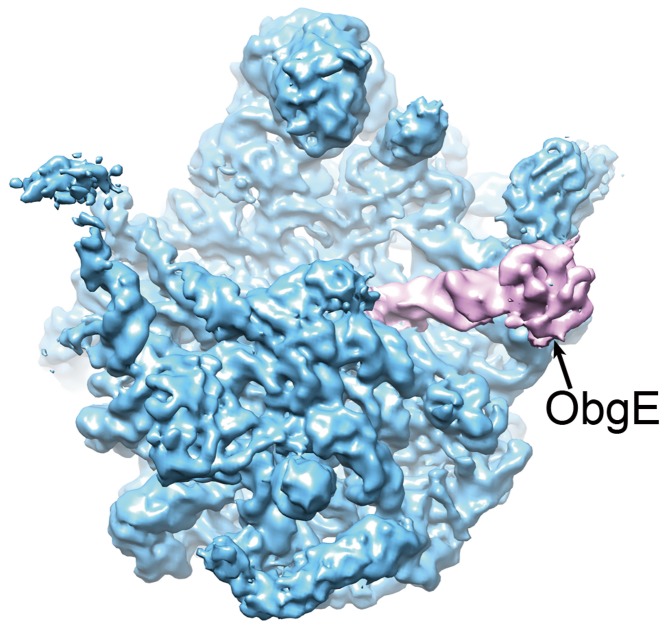
ObgE (lilac) bound to the inner surface of the large ribosomal subunit (blue), preventing its association with the small subunit (not shown). *Image Credit: Boya Feng*.

Using cryo-electron microscopy, the authors are able to discern how ObgE does this job; they find that it binds neatly to the inside face of the large subunit (Figure 1)—the very surface that the large subunit uses to associate with the small subunit. ObgE would therefore be expected to block the binding of several other GTPases with key roles in translation (IF2, EF-G, EF-Tu, RF3), as well as obstructing the enzymatic core of the ribosome—the peptidyl transfer centre. In forming this association, both partners—ObgE and the large ribosomal subunit—undergo substantial structural rearrangement to accommodate each other. From their model, the authors identify the amino acids of ObgE that are likely to interact with the ribosome, and confirm their importance by mutating them. Intriguingly, they find that the unusual ObgE N-terminus exploits a structural resemblance to tRNA, allowing it to bind to one of the main ribosomal tRNA docking sites, the A-site. As with other ribosomal GTPases, association with the large ribosomal subunit enhances ObgE's intrinsic GTPase activity, making it “switch off” by converting its GTP to GDP.

So what exactly is the point of ObgE? The authors propose that it has normal roles as a checkpoint regulator of the maturation of the large subunit, and as an overseer of the recycling of ribosomal subunits during the process of routine translation, presumably involving alternating binding of GTP and GDP, as with most GTPases. However, they also suggest that in the presence of high levels of the alternative ligand (p)ppGpp, as happens in the “stringent response”, ObgE lingers on the dissociated large subunit. This prolonged interaction would prevent the release of new subunits following maturation, and would also sequester active large subunits, impeding their reunion with small subunits during the translation cycle. Both effects would help the braking process.

Although the study focuses on the workhorse bug *Escherichia coli*, it may have implications that are closer to home; ObgE is highly conserved, with relatives across the bacterial family tree, including bacterial descendants that inhabit eukaryotic cells—mitochondria and chloroplasts. Whether our own mitochondria use their version of ObgE when they need to batten down the hatches remains to be seen.

**Feng B, Mandava CS, Guo Q, Wang J, Cao W, et al. (2014) Structural and Functional Insights into the Mode of Action of a Universally Conserved Obg GTPase.** doi/10.1371/journal.pbio.1001866

